# Development of a health literacy scale for nutrition and supplement use among pregnant and postpartum women

**DOI:** 10.7586/jkbns.26.006

**Published:** 2026-05-26

**Authors:** Mi-young An

**Affiliations:** Department of Nursing, Yeoju University, Yeoju, Korea

**Keywords:** Pregnancy, Postpartum period, Nutritional status, Dietary supplements, Health literacy

## Abstract

**Purpose:**

This study aimed to develop and validate a health literacy scale to assess nutrition and dietary supplement use among pregnant and postpartum women.

**Methods:**

Baker’s health literacy model was used as the conceptual framework. Based on a comprehensive review of domestic and international literature, 60 preliminary items were generated across four domains: vocabulary, familiarity, reading comprehension, and numeracy. After two rounds of expert content validity testing and face validity assessment, 42 items were retained. Data were collected from 315 pregnant and postpartum women between March 31 and May 11, 2021, using an online questionnaire administered through Google Forms. Item characteristics and construct validity were examined using Rasch analysis based on item response theory. Concurrent validity and reliability were also evaluated, and 23 items were retained for the final instrument.

**Results:**

Exploratory factor analysis supported the unidimensionality of the scale. The Kuder-Richardson Formula 20 coefficient for the 23-item scale was 0.87, indicating high internal consistency. The optimal cutoff score was 12.50, with a sensitivity of 0.61, a specificity of 0.66, and an area under the receiver operating characteristic curve of 0.727, indicating acceptable discriminative ability.

**Conclusion:**

The developed instrument demonstrated satisfactory criterion validity and high reliability. It effectively distinguished pregnant and postpartum women with high and low levels of health literacy related to nutrition and supplement use and may be used in clinical and community settings to assess health literacy and support tailored educational interventions.

## INTRODUCTION

During pregnancy, women undergo various physiological changes, including weight gain, hormonal fluctuations, increased cardiac output, and decreased hemoglobin levels, to facilitate the normal growth and development of the fetus [1]. Additionally, there is an increased demand for nutrients such as protein, iron, folate, vitamin D, and energy throughout the pregnancy period [2,3].

Following childbirth, women undergo changes to facilitate lactation resulting in increased requirements for nutrients such as iron, calcium, folate, vitamin A, and vitamin C [1]. Adequate intake of foods containing appropriate nutrients and energy, along with nutritional management, is crucial to support all these physiological changes [1].

However, pregnant and postpartum women often fall short of the recommended intake for minerals and vitamins [1,4]. Inadequate nutritional intake during pregnancy can lead to adverse health outcomes for the mother, such as anemia, sepsis, and preeclampsia. Furthermore, it can result in intrauterine growth restriction, preterm birth, and low birth weight for the fetus, complications that may influence the child's lifelong quality of life and healthcare expenditures [5,6]. Therefore, it is necessary to prevent adverse maternal and neonatal outcomes during the perinatal period through proper nutrition management for pregnant and postpartum women, which can influence the health of both mothers and infants [4].

When pregnant and postpartum women consider supplementing their inadequate nutrition with supplements, since the recommended dosage of nutritional supplements for pregnant and postpartum women differs from that for the general adult population, attention should be paid to proper intake [7]. Excessive intake of folic acid can promote cancer and impair fetal development under certain conditions [1]. Incorrect acquisition of information about nutritional supplements can lead to side effects such as fetal malformations and neonatal nutrient deficiencies due to misuse [1]. Therefore, education is necessary to help pregnant and postpartum women select supplements with appropriate dosages to prevent misuse [8]. To facilitate such education, it is necessary to measure pregnant and postpartum women's health literacy regarding nutrition and supplements to provide tailored education based on empirical evidence. Health literacy has a direct impact on individuals' health management and quality of life [9]. In particular, pregnant and postpartum women's health literacy influences their decisions regarding health management during pregnancy and lactation, as it involves accessing and understanding information and determining the motivation and ability to use it [10,11]. Lower health literacy increases the risk of misunderstanding health information and advice, as well as challenges in accurately adhering to prescribed medications [9,12]. Moreover, difficulty in acquiring information from various sources and finding objective information can lead to improper supplement use, posing risks to maternal and infant health [13]. Pregnant and postpartum women with higher health literacy tend to have higher rates of antenatal care, and there are differences in the health outcomes of newborns and mothers after childbirth [14,15].

Health literacy refers to the ability to read and comprehend information in healthcare environments [9]. Understanding health-related materials is more closely linked to health than general information literacy [9,16,17]. In addition, the importance of health literacy has recently been highlighted as one of the health determinants [16]. Therefore, measuring health literacy is more significant for predicting health outcomes [16,17].

While current health literacy instruments for this population address general pregnancy-related knowledge, there remains a significant gap in assessing information literacy specifically tailored to nutrition and supplement use [18]. Moreover, research on health literacy related to nutrition primarily focuses on assessing nutritional health literacy for elementary students and adults [19,20]. Among the 205 global health literacy measurement tools provided by the health literacy Tool Shed, there are still no tools specifically addressing nutrition and supplement-related health literacy for pregnant and postpartum women [21].

In clinical settings, it is beneficial to assess patients’ basic concepts, knowledge, and health literacy in order to understand their learning needs prior to implementing educational programs [17]. Therefore, assessing health literacy can be an effective method for tailoring education and counseling to individual capabilities. At this juncture, there is a need for the development of a measurement scale to assess the health literacy of pregnant and postpartum women regarding nutrition and supplement use.

This instrument was developed to serve as a screening tool to identify potential biological health risks pregnant and postpartum women at an early stage and to prevent adverse health outcomes. Therefore, this study aimed to identify the components of health literacy regarding nutrition and supplement use among pregnant and postpartum women, including both expectant and lactating mothers. Based on these components, this study aimed to develop measurement items and evaluate the reliability and validity of the resulting scale.

## METHODS

### 1. Study design

**Figure 1.**
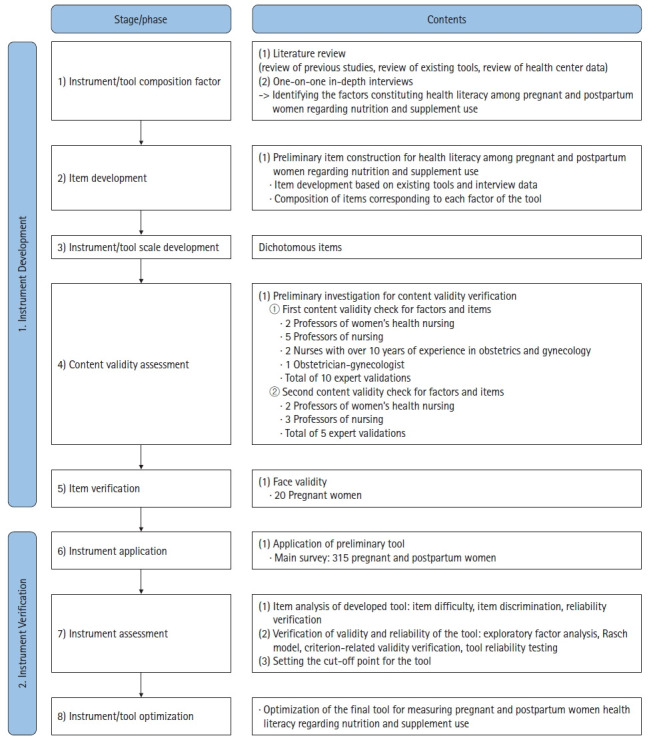
Scale development process.

This study is a methodological investigation aimed at developing a measurement tool to assess the health literacy of pregnant and postpartum women regarding nutrition and supplement use, based on the scale development process [22]. In this study, the researcher verified the validity and reliability of the developed instrument (Figure 1).

#### 1) Phase 1: scale development

##### (1) Scale composition factors

In this study, the researcher reviewed domestic and international literature to encompass the comprehensive content of health literacy regarding nutrition and supplement use among pregnant and postpartum women. Additionally, comprehensive examinations were conducted on educational materials for pregnant and postpartum women's nutrition and supplement use, including materials provided by public agencies under the Ministry of Health and Welfare, health centers, women's hospitals, and universities' portals. The researcher conducted one-on-one in-depth interviews. The researcher also reviewed health literacy measurement tools presented in the health literacy Tool Shed [21]. Data were collected from nine pregnant and postpartum women regarding commonly consumed supplements, prohibited foods, and their comprehension of nutritional terminology. Specifically, these qualitative insights allowed the researcher to identify common misconceptions and unfamiliar terms, which were then formulated into clear, context-specific test items. This ensured that the instrument accurately reflected the real-world experiences and comprehension levels of the target population.

##### (2) Establishing the item pool

This study was developed based on Baker's [17] health literacy model. The identified factors were developed according to two subdomains: prior knowledge (vocabulary and familiarity) and reading proficiency (comprehension and numeracy). To assess understanding of word meanings, the vocabulary and familiarity domain was included. All items in the developed tool are dichotomous items, measuring scores as either correct or incorrect.

##### (3) Content validity verification

To verify the accuracy of the content measurement, two rounds of content validity were conducted based on expert opinions.

In this study, a group of 10 experts was comprised, including two professors of women's health nursing, five nursing professors, two nurses with over ten years of experience in obstetrics, and one obstetrician. The first round of content validity was conducted with this group of experts. Following the first round, a second round of content validity was administered to the same group of experts for the items developed in the initial round. For the second round, two professors of women's health nursing and three nursing professors with knowledge of health literacy, totaling five experts from the first round, were consulted. Items with an item-content validity index (I-CVI) of 0.80 or higher were selected.

Face validity was confirmed by assessing the comprehensibility of the items and the overall content and format of the questionnaire with a sample of 20 pregnant and postpartum women.

#### 2) Phase 2: evaluation of the scale

##### (1) Study participants

The study participants were pregnant women, including those classified as high-risk, and women within 12 months postpartum who were able to read and understand Korean and who provided informed consent. It is suggested that a minimum of 300 participants is needed to achieve stable inter-item correlations [22]. Based on this recommendation, a target sample size of 315 participants was determined. Of the 339 women initially recruited, 21 were excluded due to missing or invalid responses, and 3 foreign nationals were excluded because their linguistic and sociocultural backgrounds were considered substantially different from those of the target population. Consequently, data from 315 participants were included in the final analysis.

##### (2) Scale/instrument evaluation

To assess the appropriateness of item difficulty and discrimination, item analysis was conducted. Item analysis involved calculating mean scores, standard deviations, and point-biserial correlations.

To assess the reliability of the developed preliminary instrument, the internal consistency of the items was measured using the Kuder–Richardson 20 (KR-20) coefficient [23].

For the validation and reliability verification of the developed instrument, exploratory factor analysis (EFA) and the Rasch model were employed. To test the content validity, the Korean Health Literacy Assessment Tool (KHLAT) [24] and short-form Korean Health Literacy Test (S-KHLT) [25] were used. To determine the measurement criteria for the final developed instrument, a receiver operating characteristic (ROC) curve analysis was performed. Sensitivity and specificity were plotted on the curve to identify the optimal cutoff point.

##### (3) Scale/instrument optimization

Based on the results of the validity and reliability testing of the instrument measuring health literacy regarding nutrition and supplement use during pregnancy and lactation, the researcher optimized the final instrument to 23 items.

### 2. Data collection

Data were collected online from pregnant and postpartum women, the target population, from March 31, 2021, to May 11, 2021. Subject recruitment was conducted through 13 institutions, including three comprehensive hospitals (one tertiary hospital, one university hospital, and one general hospital), three specialized women's hospitals, six breastfeeding clinics and postpartum care centers, and one mom cafe with a membership of 370,000. Participants were recruited by posting recruitment notices at these institutions.

### 3. Data analysis

Data were analyzed using SPSS version 25.0 (IBM Corp., Armonk, NY, USA), jMetrik 4.1.1, and SAS Version 9.4 (SAS Institute, Cary, NC, USA). Participants’ general characteristics were analyzed using means, standard deviations, frequencies, and percentages.

EFA was conducted using SPSS version 25.0 to assess whether the items met the assumptions of unidimensionality and local independence, prerequisites for item response theory (IRT) application [26]. Principal component analysis was used to examine eigenvalues, and scree plots and explained variance were reviewed to identify the dominant factor. A scale was considered unidimensional if the primary factor’s eigenvalue was substantially higher than those of other factors and accounted for at least 20% of the total variance [27].

Content validity was evaluated using the CVI and face validity. Construct validity was examined through item analysis, reliability testing, EFA, and Rasch model analysis. In the Rasch analysis, item difficulty, discrimination, model fit, item characteristic curves, test information function curves, item maps, and item functioning were assessed.

Convergent validity was evaluated using Pearson correlation coefficient. Reliability was assessed using the KR-20. To determine the optimal cutoff point and assess the diagnostic performance of the scale, ROC curve analysis was performed. The area under the ROC curve (AUROC) was interpreted based on the criteria suggested by Hanley and McNeil [28]: 0.50–0.59 (fail), 0.60–0.69 (poor), 0.70–0.79 (fair), 0.80–0.89 (good), and 0.90–1.0 (excellent). An AUROC of 0.50 was considered to indicate no diagnostic value.

Differential item functioning (DIF) was evaluated using the Mantel–Haenszel chi-square procedure in jMetrik 4.1.1 [29] to determine whether items functioned differently by occupational status. DIF occurs when individuals with equivalent ability have differing probabilities of correctly answering an item due to group characteristics [29]. Items were classified based on the common odds ratio (COR) and chi-square values: Grade A (*χ*^2^ > 0.05 or 0.65 ≤ COR ≤ 1.53), Grade C (COR < 0.53 and 95% confidence interval [CI] upper limit < 0.65), and Grade B (neither A nor C) [30]. Items favoring the focal group were denoted “+,” and those favoring the reference group were denoted “–.” In this study, participants without employment served as the reference group, and participants with employment as the focal group.

### 4. Ethical considerations

After receiving approval from the Institutional Review Board (IRB) of Gachon University (IRB No. 1044396-202008-HR-155-01), the researcher conducted the study. Participants recruited online were informed of the purpose of the study, research procedures, confidentiality, and the possibility of withdrawal during questionnaire completion. Upon obtaining their consent, the researcher proceeded with the survey.

Participants were first presented with a comprehensive information sheet detailing the study objectives, procedures, potential risks, and data privacy measures. Access to the survey items was strictly limited to those who voluntarily selected the mandatory 'I Agree' checkbox. To guarantee anonymity, no IP addresses or personally identifiable information were collected. Furthermore, participants were explicitly informed of their right to withdraw at any point during the survey without any penalty by simply closing the browser.

## RESULTS

### 1. Phase 1: scale development

Based on the in-depth interviews and literature review, the researcher developed the health literacy construct for pregnant and postpartum women's nutrition and supplement use, focusing on reading proficiency (comprehension and numeracy) and prior knowledge (vocabulary and familiarity). A preliminary pool of 60 items was created, with 34 items related to vocabulary and familiarity and 26 items related to comprehension and numeracy. The developed instrument was structured as dichotomous data, with correct answers scored as 1 and incorrect answers as 0.

Content validity of the instrument was evaluated through two rounds of expert review. In the first round, the I-CVI for the 60 preliminary items ranged from 0.80 to 1.00, with a scale-level average (S-CVI/Ave) of 0.97, indicating strong content validity. Based on expert feedback regarding redundancy and nutritional irrelevance, 13 items were deleted and 8 items were revised, resulting in a 47-item pool.

In the second round, five experts evaluated the 47-item pool. The I-CVI for individual items ranged from 0.80 to 1.00, and the S-CVI/Ave was 0.99, demonstrating excellent content validity. Following further expert recommendations, 5 items were deleted and 6 items were revised, producing a final 42-item instrument, including 18 items assessing vocabulary and familiarity and 24 items assessing comprehension and numeracy.

Following the expert content validity assessment, the researcher conducted face validity with 20 pregnant and postpartum women to confirm the comprehensibility and suitability of the items and questionnaire format. After confirmation without further modification, the final preliminary tool was completed with 42 items.

### 2. Phase 2: evaluation of the scale

In the second phase/stage, 315 pregnant and postpartum women participated. The characteristics of the participants are shown in Table 1.

#### 1) Analysis of item of the developed instrument/ scale

Item analysis was conducted to identify items with appropriate difficulty and discrimination. Of the 42-item preliminary scale, 24 items met the KR-20 criterion of 0.89, indicating adequate internal consistency. These 24 items were retained for further analysis.

#### 2) Instrument validity and reliability assessment

EFA was conducted to verify that the 24 selected items satisfied the assumptions of unidimensionality and local independence, prerequisites for applying IRT. Both assumptions were met. The eigenvalue of the first factor was 6.73, which was substantially higher than those of the other factors, and it accounted for 28.05% of the total variance, thereby demonstrating unidimensionality.

Rasch analysis was performed to assess model and item fit. Item difficulty ranged from −1.33 to 2.84. All items demonstrated appropriate difficulty, except for item 15 (neural tube defects), which was retained due to its clinical significance in maternal nutrition.

Item discrimination was evaluated using point-biserial correlation coefficients, which ranged from 0.30 to 0.68. Item 25 exhibited low discrimination (0.23) and was removed. Consequently, the final scale consisted of 23 items. All remaining items had discrimination coefficients ≥ 0.30 and no negative values, indicating adequate discriminative ability (Table 2). Item fit was evaluated using infit and outfit indices. Infit values ranged from 0.70 to 1.37, and outfit values ranged from 0.51 to 1.93. Infit and outfit values between 0.5 and 2.0 are acceptable, with values closer to 1 indicating that items adequately reflect the underlying construct. Items with fit values above 2.0 are considered misfitting [31]. Therefore, the fit indices of all 24 items met the evaluation criteria, indicating good model fit. This suggests that the items reliably measure health literacy and can be applied to identify pregnant and postpartum women who may require targeted educational interventions. Considering difficulty, discrimination, and fit, a final set of 23 items was retained (Table 2). DIF was analyzed using occupational status as a grouping variable. All 23 items were classified as Grade A, indicating no DIF.

Concurrent validity was evaluated by correlating the developed instrument with existing tools. The correlation with KHLAT was 0.64 (*p* < .001), indicating strong concurrent validity, whereas the correlation with S-KHLT was 0.37 (*p* < .001), indicating moderate validity (Table 3).

Reliability analysis of the final 23 items yielded a KR-20 coefficient of 0.87, with item-level KR-20 values ranging from 0.86 to 0.87, demonstrating high internal consistency (Table 4).

#### 3) Instrument/ scale cutoff point setting

The developed tool in this study consists of a total of 23 items with a score range of 0 to 23 points. To ensure the utility of the developed tool, the cutoff point was measured. The cutoff point of the test showed a cross point at 12.50 points with a sensitivity of 0.61 and specificity of 0.66. Therefore, a score of 13 points or higher indicates an appropriate level of health information literacy, while a score of 12 points or lower suggests an inadequate level of health information literacy. The diagnostic accuracy of the test tool, represented by the AUROC, was 0.727 (95% CI, 0.62–0.82).

#### 4) Instrument/ scale optimization

Based on the results of the validity and reliability testing, the health literacy measurement tool for pregnant and postpartum women regarding nutrition and supplement use was refined to a final set of 23 items. The instrument consists exclusively of objective, performance-based items designed to assess participants’ knowledge, comprehension, and numeracy related to nutrition and supplement use.

Of the 23 items, 15 assess vocabulary and familiarity with terms commonly encountered during pregnancy and the postpartum period. The remaining 8 items evaluate comprehension and numeracy skills, including knowledge of folic acid and iron supplementation, dietary recommendations for pregnant and postpartum women, appropriate supplement selection, interpretation of hemoglobin levels, and alcohol consumption during pregnancy.

All items are dichotomously scored (correct = 1, incorrect = 0), yielding a total possible score of 23. A cutoff score of 13 was established to indicate a high level of health information literacy.

## DISCUSSION

The health literacy measurement scale for pregnant and postpartum women's nutrition and supplement use was developed to objectively measure individuals' capacity to understand and utilize health information for the promotion of maternal health, disease prevention, and ultimately the health promotion and disease prevention for fetuses and infants.

This scale was developed using educational materials on nutrition and supplements for pregnant and postpartum women, as well as supplement usage manuals. Utilizing materials experienced by pregnant and postpartum women in their daily environment enhances the familiarity of the subjects with the test, increases voluntary participation, and interest, thereby reducing test dropout rates. Additionally, it can be considered a tool that takes into account the cognitive level of pregnant and postpartum women, the health information environment, and practicality.

The average time required for measurement is short, at 10.89 minutes, making it efficient. No separate training is required for the examiner when applying the developed tool. Participants also do not require education or preparation, and since scoring and response are measured by correct and incorrect answers, scoring and response are simple, allowing for group testing. Furthermore, by testing the validity of the tool through item response theory, it reflects the unique characteristics of the items and enables an objective estimation of the participants' abilities. Presenting the cutoff point of the tool proves its utility in identifying individuals with low health literacy.

The ability to communicate orally about health information, known as oral health literacy, is deeply intertwined with the cognitive processes required for understanding words [17]. Therefore, in this study, the researcher developed the vocabulary and familiarity domains, which are precursory knowledge areas, together. Similar to the vocabulary and familiarity aspects of this tool, the Rapid Estimate of Adult Literacy in Medicine [32] focuses on measuring the ability to read words. However, the difference lies in the fact that the tool developed in this study measures prior knowledge regarding the meanings of words accurately.

The KHLAT [24], like the instrument in this study, assesses whether participants understand the meanings of specific terms. However, while KHLAT [24] measures general medical terminology used in clinical settings, the tool developed in this study reflects the unique needs of pregnant and postpartum women by focusing on nutrition and supplement-related terms. The correlation with S-KHLT [25] was moderate (r = 0.37, *p* < 0.001), which is relatively lower than that of KHLAT. This discrepancy can be attributed to the conceptual differences in the scope of measurement. While S-KHLT assesses general health literacy, focusing primarily on formal reading comprehension and navigating the healthcare system, the developed instrument is highly specialized for functional and interactive literacy related to maternal nutrition. Specifically, S-KHLT measures broad medical literacy, whereas this tool evaluates domain-specific knowledge and decision-making skills essential for pregnancy-related physiological needs. These results suggest that while the new instrument aligns with the overarching construct of health literacy, it possesses distinctive incremental validity by capturing unique nutritional aspects that general tools like S-KHLT might overlook.

In the domain of reading proficiency, comprehension and numeracy, the Test of Functional Health Literacy in Adults [33] and S-KHLT [25] measure contents such as instructions and prescriptions commonly used in hospitals. The Newest Vital Sign [34] measures comprehension and numeracy using ice cream labels. The tool developed in this study measures comprehension and numeracy by including questions related to the intake of folic acid and iron supplements, nutritional intake standards for pregnant and postpartum women, information regarding nutritional supplement choices, hemoglobin levels, and alcohol consumption during pregnancy and postpartum. Using measurement tools that are highly likely to be encountered by patients feels more comfortable and natural for the participants than using other tools for academic measurement [17]. Therefore, this tool is acceptable to participants as it uses familiar content that pregnant and postpartum women encounter in their daily lives.

Maternal Health Literacy Inventory for Pregnancy [18] is a tool developed to measure knowledge regarding prenatal care among pregnant women. As a subjective self-report assessment tool, it is difficult to objectively compare the difficulty of questions or the level of ability among participants. In contrast, the tool developed in this study measures health literacy rather than knowledge. Moreover, it was developed using the Rasch model of item response theory, which allows for the objective measurement of participants' abilities by excluding subjectivity resulting from environmental influences.

Nutrition Literacy Assessment Instrument (NLit) [30] and Self-Perceived Food Literacy (SPFL) [35] are both tools for measuring nutritional literacy. NLit [30] measures the ability to read nutrition-related terms and labels and assesses correctness. SPFL [35] measures skills related to food preparation, healthy snacking styles, and food labeling. While both tools can measure adults' understanding of diet and nutrition, measuring health literacy specifically related to nutrition is challenging. The tool developed in this study was designed to measure health literacy specifically related to nutrition and was developed for pregnant and postpartum women, distinguishing it from existing tools.

Upon reviewing the excluded 19 items out of the initially developed preliminary tool of 42 items, it was found that questions related to nutrition information and supplement intake methods for iron and folic acid were almost entirely eliminated due to high scores. Similarly, questions concerning safe dietary habits for pregnant and postpartum women, such as food poisoning and water intake, were also deleted as they obtained high scores. This is believed to be because pregnant and postpartum women are familiar with these topics as they encounter them frequently in their daily lives. Additionally, it is thought that the young age range (20s to 40s) and high level of education among the participants may have influenced these results. Based on these findings, it is suggested that adjusting the difficulty level of items in future item development should consider characteristics such as age and education level of the participants.

Although Rasch analysis indicated good overall model fit, several limitations should be considered. First, the sample was predominantly women in their 30s with university-level education, with only 11.3% having a high school education or lower. This demographic skew may have resulted in a ceiling effect, potentially underestimating the difficulty of basic literacy items and masking misfitting items due to high response consistency among highly educated participants. In addition, prior knowledge and literacy levels may vary significantly based on specific maternal characteristics, such as high-risk pregnancies, primiparity, or multicultural backgrounds, which were not fully represented in this sample.

Furthermore, the data were collected between March and May 2021, which may limit the direct applicability of the findings to current clinical and research contexts. Changes in health information environments and patterns of nutrition and dietary supplement use over time may influence health literacy levels. Consequently, the findings from the Rasch analysis should be interpreted with caution, and future studies are needed to validate the instrument in more diverse and recent populations including younger, less educated, or high-risk pregnant and postpartum women to ensure the stability, generalizability, and applicability of item parameters.

## CONCLUSION

Based on the results of this study, the health literacy measurement scale for pregnant and postpartum women's nutrition and supplement use has been confirmed as a valid and reliable instrument. This study suggests the potential of using it as a standard for conveniently measuring pregnant and postpartum women's health literacy in clinical and public health settings and providing education and interventions tailored to the abilities of the participants.

## Figures and Tables

**Table 1.** General Characteristics of the Participants (N = 315)

Characteristics	Categories	n (%)	Mean ± SD
Age (years)	22–29	55 (17.5)	33.81 ± 4.34
	30–39	225 (71.4)	
	40–46	35 (11.1)	
Education	High school	36 (11.4)	
	Associate degree	83 (26.4)	
	Undergraduate	172 (54.6)	
	Graduate	24 (7.6)	
Working status	No	145 (46.0)	
	Yes	170 (54.0)	
Breastfeeding (months)	1–3	100 (31.7)	5.96 ± 3.05
	4–6	51 (16.2)	
	7–12	72 (22.9)	
	None	92 (29.2)	
Knowledge level of nutrition and supplements	Know very well	11 (3.5)	
	Know well	50 (15.9)	
	Moderate	187 (59.3)	
	Barely know	62 (19.7)	
	No idea	5 (1.6)	
Nutrition and supplement intake education experience	Yes	62 (19.7)	
	No	253 (80.3)	
Number of education experiences	1 time	37 (11.8)	
	2 times	18 (5.7)	
	3 times or more	8 (2.5)	
	None	252 (80.0)	
Ability to read guidebooks	1–3	90 (28.6)	5.27 ± 2.43
	4–5	73 (23.1)	
	6–8	131 (41.6)	
	9–10	21 (6.7)	
Level of understanding of guidebooks	1–3	45 (14.3)	6.12 ± 2.17
	4–5	68 (21.6)	
	6–8	166 (52.7)	
	9–10	36 (11.4)	
Degree of nutrition and supplement study	1–3	60 (19.1)	5.73 ± 2.25
	4–5	76 (24.1)	
	6–8	151 (47.9)	
	9–10	28 (8.9)	
How to obtain information during pregnancy	Family	21 (6.7)	
	Friend or colleague	21 (6.7)	
	Online mothers’ community, Blog	110 (34.9)	
	Internet search (Google, Naver, etc.)	97 (30.8)	
	Experts (doctors, nurses, pharmacists)	56 (17.8)	
	Books/Magazines	8 (2.5)	
	Mass media	2 (0.6)	
How to obtain information after delivery	Family	24 (7.6)	
	Friend or colleague	28 (8.9)	
	Online mothers’ community, Blog	115 (36.5)	
	Internet search (Google, Naver, etc.)	95 (30.1)	
	Experts (doctors, nurses, pharmacists)	44 (14.0)	
	Books/Magazines	5 (1.6)	
	Mass media	4 (1.3)	

Scores ranged from 1 to 10, with higher scores indicating better comprehension. SD = Standard deviation.

**Table 2.** Item Difficulty, Discrimination, and Suitability (N = 315)

No.	Item contents	Difficulty (Mean ± SD)	Discrimination	Suitability	
			Point-biserial correlation	Infit	Outfit
1	Do you understand the definition of protein?	-0.03 ± 0.13	.54	0.86	0.81
2	Do you understand the definition of a calorie?	-0.48 ± 0.14	.54	0.86	0.76
3	Do you understand the definition of anemia?	-0.83 ± 0.14	.59	0.79	0.65
4	Do you understand the definition of iron?	-0.57 ± 0.14	.62	0.74	0.60
5	Do you understand the definition of folic acid?	0.53 ± 0.13	.57	0.86	0.78
6	Do you understand the definition of vitamin D?	-0.28 ± 0.14	.62	0.78	0.67
7	Do you understand the definition of multiple pregnancy?	0.17 ± 0.13	.48	1.06	1.01
8	Do you understand the definition of pre-eclampsia?	0.79 ± 0.13	.58	0.87	0.78
9	Do you understand the definition of gestational diabetes?	0.02 ± 0.13	.63	0.81	0.71
11	Do you understand the definition of premature birth?	-1.12 ± 0.15	.68	0.70	0.51
12	Do you understand the definition of premature rupture of membranes?	1.45 ± 0.14	.58	0.82	0.68
15	Do you understand the definition of a neural tube defect?	2.84 ± 0.18	.35	0.87	1.10
16	Do you understand the definition of a low-birth-weight infant?	-0.52 ± 0.14	.59	0.85	0.73
17	Do you understand the definition of intrauterine growth retardation?	1.36 ± 0.14	.50	0.91	0.83
18	Do you understand the definition of fetal alcohol syndrome?	1.72 ± 0.15	.47	0.88	0.70
22	If I drank a glass of milk at 6 a.m., what time should I take an iron supplement?	-1.08 ± 0.15	.31	1.33	1.40
23	What can I take with iron supplements to enhance absorption?	0.16 ± 0.13	.38	1.25	1.26
24	How many additional Kcal should be consumed per day during mid-pregnancy?	-1.12 ± 0.15	.30	1.32	1.40
26	What complications can occur in babies if there is a lack of folic acid in early pregnancy?	-1.33 ± 0.16	.48	0.98	1.14
28	How many milligrams of folic acid supplementation are recommended from preconception to at least 12 weeks of pregnancy?	-0.10 ± 0.13	.33	1.33	1.65
31	Here are the results of your hemoglobin test. Is your hemoglobin level within the normal range?	-1.05 ± 0.15	.30	1.35	1.38
32	What symptoms may occur in a baby if a pregnant woman drinks heavily during early pregnancy?	-0.67 ± 0.14	.44	1.10	1.35
35	If a breastfeeding mother drinks a beer at 5 p.m., when can she breastfeed her baby?	-1.30 ± 0.15	.42	1.09	1.53

SD = Standard deviation.

**Table 3.** Criterion Validity of the Developed Scale (N = 315)

Variables	Developed scale	KHLAT	S-KHLT
	*r* (*p*)		
KHLAT	.64 (< .001)	1	-
S-KHLT	.37 (< .001)	.27 (< .001)	1

KHLAT = Korean Health Literacy Assessment Tool; S-KHLT = Short Form of Korean Functional Health Literacy Test.

**Table 4.** Reliability of the Scale (N = 315)

Item	Item contents	KR-20 if item deleted
1	Do you understand the definition of protein?	.86
2	Do you understand the definition of a calorie?	.86
3	Do you understand the definition of anemia?	.86
4	Do you understand the definition of iron?	.86
5	Do you understand the definition of folic acid?	.86
6	Do you understand the definition of vitamin D?	.86
7	Do you understand the definition of multiple pregnancy?	.87
8	Do you understand the definition of pre-eclampsia?	.86
9	Do you understand the definition of gestational diabetes?	.86
11	Do you understand the definition of premature birth?	.86
12	Do you understand the definition of premature rupture of membranes?	.87
15	Do you understand the definition of a neural tube defect?	.87
16	Do you understand the definition of a low-birth-weight infant?	.86
17	Do you understand the definition of intrauterine growth retardation?	.87
18	Do you understand the definition of fetal alcohol syndrome?	.87
22	If I drank a glass of milk at 6 a.m., what time should I take an iron supplement?	.87
23	What can I take with iron supplements to enhance absorption?	.87
24	How many additional Kcal should be consumed per day during mid-pregnancy?	.87
26	What complications can occur in babies if there is a lack of folic acid in early pregnancy?	.87
28	How many milligrams of folic acid supplement are recommended from preconception to at least 12 weeks of pregnancy?	.88
31	Here are the results of your hemoglobin test. Is your hemoglobin level within the normal range?	.87
32	What symptoms may occur in a baby if a pregnant woman drinks heavily during early pregnancy?	.87
35	If a breastfeeding mother drinks a beer at 5 p.m., when can she breastfeed her baby?	.87
	Total KR-20 reliability = 0.87	

KR-20 = Kuder Richardson-20.
